# Interleukin-7 and immune reconstitution in cancer patients: a new paradigm for dramatically increasing overall survival

**DOI:** 10.1007/s11523-012-0210-4

**Published:** 2012-03-02

**Authors:** Michel Morre, Stéphanie Beq

**Affiliations:** Cytheris S.A., 175 rue Jean Jacques Rousseau, 92130 Issy-Les-Moulineaux, France

**Keywords:** IL-7, Immunotherapy, Cancer, Immune reconstitution

## Abstract

Although great effort is being expended in the development of cancer immunotherapies, it is surprising that global lymphopenia and its various dimensions are not being systematically assessed in cancer patients. The incident pathologies associated with various immunosuppressed conditions such as those found in HIV infection have taught us that measuring various T cell populations including CD4 provides the clinician with a reliable measure for gauging the risk of cancer and opportunistic infections. Importantly, recent data emphasize the key link between lymphocyte T cell counts and overall survival in cancer patients receiving chemotherapy. Treatment of immunocompromised patients with interleukin-7 (IL-7), a critical growth and homeostatic factor for T cells, has been shown to produce a compelling profile of T cell reconstitution. The clinical results of this investigational therapy confirm data obtained from numerous preclinical studies and demonstrate the long-term stability of this immune reconstitution, not only on CD4 but also on CD8 T cells, involving recent thymic emigrants as well as naive, memory, and central memory T cells. Furthermore, IL-7 therapy also contributes to restoration of a broadened diversity of the T cell repertoire as well as to migration of these cells to lymph nodes and tissues. All these properties support the initiation of new clinical studies aimed at reconstituting the immune system of cancer patients before or immediately after chemotherapy in order to demonstrate a potentially profound increase in overall survival.

## Introduction

Numerous articles have been devoted to reviewing the immune properties of interleukin-7 (IL-7) and this molecule's potential for therapeutic use. The most recent of these articles provides a comprehensive review of these therapeutic avenues along with the supportive immune rationale [1]. Taking a more focused approach, we here intend to delve into the specific problem of lymphopenia in the oncology patient, first analyzing its major aspects and pathological consequences and then demonstrating how recombinant IL-7 represents a potentially effective therapy for treatment of this condition and a solution to a longstanding medical problem.

## The immune status of cancer patients: poorly monitored and often ignored

Although it might seem obvious that tracking immune system status is crucial to inhibiting carcinogenesis and maintaining cellular homeostasis [2], many cancer patients are surprisingly more immunocompromised than suspected by their treating physician. In fact, with the exception of patients receiving hematopoietic stem cell transplants (HSCT) for treatment of hematologic malignancies, the immune status of cancer patients is rarely evaluated and when attempted, is frequently done poorly.

When oncologists do measure absolute lymphocyte counts, they do not usually evaluate T cell (CD3) numbers, rarely measure T cell helpers (CD4), and only exceptionally, evaluate the other T cell subpopulations and the breadth of the T cell receptor (TCR) repertoire. As a result, until recently, the potential consequence of lymphopenia and its association with overall patient survival was poorly documented. In fact, this is often the case when a biomarker is not expected to change with an approved therapeutic intervention. If neutrophil counts are monitored, it is partly due to the fact that corrective measures can be undertaken through G-CSF administration with the same being true for platelets with blood transfusion and red blood cells utilizing therapy with erythropoietin.

Although the deficit of specific or local anti-tumor response had been intensively explored, prior to 2003 most data aimed at evaluating the global immune deficit of cancer patients came from Mackall's team in the USA and the Blay/Ray-Coquard's team in France. In two landmark papers, Mackall et al. [3, 4] explored the immune reconstitution of young patients who had undergone intensive chemotherapy for cancer. In their published studies, the Mackall team demonstrates that CD4 T cell reconstitution occurs primarily in children while young adults exhibit deficiencies in this pathway. This suggests that rapid T cell regeneration requires residual thymic function in these patients, while CD8 recovery is much faster and less dependent of thymic activity. Between 1996 and 2004, Blay et al. conducted various studies demonstrating a relationship between early lymphopenia (absolute lymphocyte count (ALC) <700/mm3) from days 1 to 5 following chemotherapy and the risk of febrile neutropenia. In another study, they were also able to identify a direct link between CD4 lymphopenia, febrile neutropenia, and early death after cytotoxic chemotherapy [5–7]. Since lymphopenia-inducing cytotoxic drugs are the mainstay of cancer treatment, a better understanding of the immune system's ability to restore a depleted T cell pool is clearly of critical importance.

## The various aspects of lymphopenia: a multi-dimensional problem

### Cell counts and intensity of response

The combined use of monoclonal antibodies and fluorescence-activated cell sorting now allows for easy tracking of changes in peripheral blood T cells and enables the quantification and analysis of immune deficit or immune reconstitution by cell function (helpers and cytotoxic T lymphocytes (CTLs)), recent history (RTEs, naïve, and memory), or status (functional, activated, or exhausted). In the clinical setting, these convenient tools are used as key biomarkers in following the immune status of patients and the effects of drug therapy. Due in part to greater awareness of the importance of measuring CD4 levels as a consequence of the HIV epidemic, absolute CD4 lymphocyte counts though readily obtained by the clinician are not often requested by oncologists. Multiple large cohort studies of HIV patients have documented the medical value of tracking CD4 T cell counts for monitoring health status. These studies clearly indicate that only those patients with CD4 counts above 500/μl have a life expectancy comparable to the HIV naive population [8–10]. And, in a study which holds great significance for cancer patients, the stratification of these HIV patients according to their CD4 count demonstrates a clear link with the incidence of AIDS and non-AIDS-related malignancies [11].

### Cell variety and coordination of response

Unlike anemia, immune deficit is not a single-cell-type deficit and thus requires a full evaluation encompassing the measurement of T and B cell counts as well as the main subpopulations including naive, effector, memory, and central memory cells along with the biomarkers of their functionality. Each subpopulation has a specific function and it is the coordination of these functions that makes up the immune response. A deficit in a specific subpopulation will abrogate the response. Investigations of HIV disease have demonstrated the importance of CD4 help and vaccine studies have emphasized the role of long-term central memory T cells. Other T cells like Tγδ and NKT also appear critical for triggering or enhancing anti-tumor responses, an attribute primarily associated with their ability to articulate innate and adaptive immunity.

The distribution of T cell subpopulations differs substantially between patients with and without cancer, increasingly so at more advanced stages of disease and in those patients who have undergone treatment. Heavily treated patients show a relative shrinkage of the naïve and central memory T cell populations but with increased proliferation and differentiation in the direction of effector memory T cells, a state described as T cell exhaustion [12].

The complete coordination of the immune response requires the production and homeostasis of these subpopulations indicating that a good immune reconstitution must encompass most if not all of these cell phenotypes.

### Cell polyclonality and breadth of response

B and T cells represent a vast array of immune competent cells, each with a specific receptor (BCR or TCR) restricting its activation to a specific antigen presented in the context of MHC molecules. This specificity implies that to confer sufficient immune protection against infectious agents and malignant cells, a T cell population must include enough diversity in the TCR repertoire to ensure a broad range of response to various pathogenic antigens. This is particularly important in controlling viruses and malignant cells which have the ability to mutate and thereby escape control of the immune system. Similarly, in the treatment of cancer patients, tumors are known to escape control of the immune system by mutation. For example, under a concept known as immunoediting [13] which is directed towards tumor elimination, the relative level of control by or escape from T cells is determined by the relative pressure from the immune system. Not surprisingly, the broader the repertoire of T cells the better the control.

Through modern PCR-based technologies, it is now possible to measure the diversity of the TCR repertoire. ImmunID Technologies (Grenoble, France), a company focused on the development of this technology, has proposed the word “divpenia” to refer to the low diversity of the TCR observed in pathologies such as those represented by chronic viral disease and cancer [14]. The company has accumulated data showing that although divpenia is frequently associated with low CD4 or CD3 counts, this is not a systematic relationship and patients with reasonable T cell counts can present with a very narrow repertoire, most likely reflecting insufficient thymic function. Interestingly, in various cohorts of HIV patients, when the latest CD4 count does not appear as a good prospective or retrospective indicator of patient health status, the nadir CD4 count clearly reflects this status, indicating the extension of past thymic and mucosal damage produced by HIV infection [15].

The extensive literature on cancer immunoediting supports the importance of analyzing the breadth of T cell TCR repertoire and the inherent breadth of T cell responses and control. This also emphasizes the need for a therapeutic agent able to protect and/or regenerate thymic functions.

## The local deficit in the deep compartment and the need for trafficking in lymphoid cells

Although counts and analysis of peripheral T cells can reflect the status of T cells in the deeper lymphoid organs and tissues, this is not a systematic relationship. Many of the T cell dysfunctions seen in liver biopsies of chronic hepatitis B virus (HBV) and hepatitis C virus (HCV) patients are not readily discernible in peripheral T cells. In HIV infection, it took almost 20 years for the medical community to understand the significance of severe lymphopenia in the gut and its key role in the persistence of T cell activation [16, 17]. Even more recently, fibrosis in the lymph nodes of HIV patients has been shown to be connected to poor immune reconstitution [18].

The immune system is largely composed of mobile cells which need to traffic and interact in order to recognize pathogenic antigens, emit an elimination message, trigger a response, and then target and eliminate the pathogen. The coordination and final outcome of these cellular functions implies the trafficking of cells from the pathogen recognition site to the lymphoid organ and back to the pathogen elimination site. Homing of T cells to lymphoid organs, tissues, and tumors requires the critical expression of chemokines and integrins. In the absence of this expression, T cells will not migrate according to their programmed functions and the immune response will stop due to a lack of good trafficking to condition their interactions and coordinate the sequence of their functions.

These data seen in the context of chronic viral infection have considerable consequences for cancer treatment and serve to further emphasize the need to assess the “local lymphopenia” within the tumor or its immediate vicinity. Neutralization of TGFβ to control fibrosis, which blocks access to the tumor, is the key to driving the anti-tumor immune response and providing T cells full access to malignant cells. Multiple preclinical models have demonstrated that T cell infiltration is critical to tumor elimination, including T cell mucosal infiltration in the area surrounding the tumor.

Due to the variety of T cell populations and functions, lymphopenia is not a single-dimensional problem. Accordingly, immune reconstitution should encompass all these dimensions in order to guarantee the intensity, breadth, rapidity, and stability of the immune responses as well as the quality of immune reconstitution.

## The critical consequences of lymphopenia in assessment of cancer risk and prognosis

### Lymphopenia and an increased risk of cancer

Clinical conditions associated with immune deficit show that this condition is indicative for patient health status and incidence of malignancies. In a landmark meta-analysis study, Grulich et al. compared the incidence of cancers in two populations of immunosupressed patients, HIV-infected patients, and the transplant recipients [19]. The study showed an increased incidence and similar distribution pattern of cancers in both populations, underscoring the high incidence of cancers known to have an infectious cause: the three AIDS defining malignancies, all HPV-related, Hodgkin's lymphoma, liver, and stomach cancers [19]. The authors go on to conclude that it is immune deficiency rather than any other risk factor that drives the incidence of cancer in these two populations.

With more than 10 years of experience in treating HIV patients with highly active antiretroviral therapy (HAART), a therapy which affords recovery of CD4 T cell counts in most patients after 2 to 4 years of treatment (>60%), figures now also show that HIV patients have an increased risk of non-AIDS defining malignancies (NADM), most likely due to the poor quality of their immune reconstitution. This was recently illustrated in a cohort study by Dauby et al. who found that age (>45) and nadir CD4 counts (<200/mm^3^) are associated with an increased incidence of NADM [20].

### Lymphopenia and poor overall survival in cancer patients

Oncologists have now started to more systematically measure ALC and to explore the link with disease progression. In 2009, Ray-Coquard et al. published a multi-cohort analysis of patients with breast cancer (BC), soft tissue sarcoma (STS), and non-Hodgkin lymphoma (NHL). The study found that lymphopenia (ALC <1,000/μl/L) was present in approximately 25% of the patients and was associated with markers of disease progression and poor overall survival: median 10 versus 14 months in BC, 5 versus 10 months in STS, and 11 versus 94 months in NHL [21]. The authors conclude that lymphopenia is an independent prognostic factor for overall progression-free survival in several cancers.

In 2010, Porrata et al. studied the relapse rate of 149 Mayo clinic patients with diffuse large B cell lymphoma treated with rituxan, cyclophosphamide, adrimamycine, vincristine, and prednisone [22]. Relapse was associated with lower ALC (1,430 vs 670 cells/mm^3^) and patients with ALC >960 cells/mm^3^ had a cumulative incidence of relapse of 6% versus 79% with an ALC <960.

In 2011, Cézé et al. conducted a retrospective study on the influence of lymphopenia as a prognostic marker of colorectal cancer in patients receiving chemotherapy [23]. Among the 260 patients analyzed, lymphopenia (ALC <1,000cells/mm^3^) appeared as an independent factor for hematological toxicity and was associated with shorter overall survival (median 16 vs. 24 months).

At the recent ASCO meeting, three abstracts showed the association of lymphopenia with a poor outcome: Lim et al. [24] poor overall survival in stage IV advanced gastric cancer; Desposorio et al. [25] faster progression of preoperative breast cancer; and, Capellino et al. [26] poor survival in locally advanced breast cancer. In another set of data, Peron et al. [27] presented the significant association of various T cell counts (CD3, CD4, and CD8) observed prior to initiation of chemotherapy with survival in lymphoma, breast cancer, and sarcoma.

This encouraging result obtained on ALC or T cell counts led the Blay/Ray-Coquard team to further document CD4 T cell counts in various populations of cancer patients, including breast cancer, and to further explore the diversity of the TCR repertoire in collaboration with ImmunID Technologies. An interim analysis of a prospective breast cancer cohort currently under evaluation was presented at the 2011 AACR meeting, indicating a striking difference in the 9-month survival rate of patients with more than 450 CD4/mm^3^ or more than 20% diversity of their TCR repertoire (83% for CD4 >450/mm^3^ vs 45% for CD4 <450/mm^3^ and 78% for TCR diversity >20% vs 17% for TCR diversity <20%) (AACR 2011 abstract 165).

All these interesting observations shed new light on cancer-related hemato-toxicity and its impact on overall survival of cancer patients, further emphasizing the protective role of the immune system in fostering the anti-infective and anti-malignant aspects of immune surveillance.

### Lack of T cell intra-tumoral infiltration: lymphopenia and dysfunction associated with poor outcome

In 1984, Miwa was the first to review the data linking cancer prognosis with tumoral T cell infiltration, indentifying the enumeration of tumor-infiltrating lymphocytes (TIL) as a good prognostic biomarker [28]. The local availability of the family of LEU and OKT monoclonal antibodies explains the interest of Japanese teams in this marker [29, 30]. Since that time, the topic has been broadly documented and continuously reviewed [31–34], the case of colorectal cancer perhaps being one of the best documented. Presented in a landmark paper in 1995, Pages et al. established the association between increased survival and a high level of infiltrating memory T cells (CD45RO+), an absence of signs of early metastatic invasion, and a less advanced pathological state [35]. In more recent papers, an effort is made to better document the various phenotypes of the TILs, emphasizing the need to document their functional status.

Among TILs the ratio of suppressor FoxP3 regulatory T (Treg) cells over their agonist CD8 or CD4 counterparts is critical to delineating the prognosis which is linked to the number of cells able to express or support a CTL activity [36, 37]. Also of interest is tracking the known “protein programmed death 1” (PD1) expressed on activated T cells. The engagement of the programmed death ligand 1 (PD-L1)/B7H1 within tumor cells or other host-derived cells results in the down regulation of T cell function and represents an important negative regulatory pathway [38, 39].

## IL-7 for immune reconstitution: preclinical data

IL-7 was identified as a human T cell growth factor in 1987 [40]. The origin of circulating IL-7 is not completely elucidated as it can apparently originate from various organs. What is definitively known is that it is produced in lymphoid organs by thymic hepithelial cells, bone marrow stromal cells and, in non-lymphoid organs, by liver and intestinal epithelial cells, keratinocytes, and fibroblasts. In lesser amounts, dendritic cells and macrophages can also produce IL-7 (see for a review [41]). In solid tissue, IL-7 is bound/presented at the surface of T cells by fibronectin and heparin sulfate [42]. The IL-7 receptor is only present on the cell surfaces of hematopoietic lineage, including T cells, dendritic cells, macrophages, and NK subsets. Recently, the known biology of IL-7 has been well summarized in an excellent review by Fry and Mackall [1], allowing us here to confine ourselves to emphasizing a few points seen as critical to the therapeutic use of IL-7.

### IL-7 is key for T cell production and homeostasis

IL-7 is a non-redundant cytokine essential for T cell production. Indeed, IL-7/IL-7 R gene knockout (KO) mice do not produce mature T cells [43, 44]. In these models, the thymocytes are blocked at an early stage of differentiation and thymus cellularity is dramatically reduced. In vitro studies showing the essential role of IL-7 in establishing a competent immune system in humans [45] were confirmed by a few SCID (severe combined immunodeficiency disease) syndrome cases [46–49]. These patients presented a profound T cell deficiency due to a defect in the IL-7 receptor leading to an incapacity for binding to the cytokine or for signal transduction. IL-7 is also a major player in the regulation of peripheral T cell homeostasis [50], as demonstrated in 2001 by Fry et al. [51] and Napolitano et al. [52] who show the inverse relationship between endogenous blood levels of IL-7 and counts of CD4 T cells in HIV-infected patients, a feedback regulation already documented for erythropoietin in anemic patients. Furthermore, IL-7 is also implicated in the survival, proliferation, differentiation, and metabolism of peripheral T cells [53, 54].

**Fig. 1:**
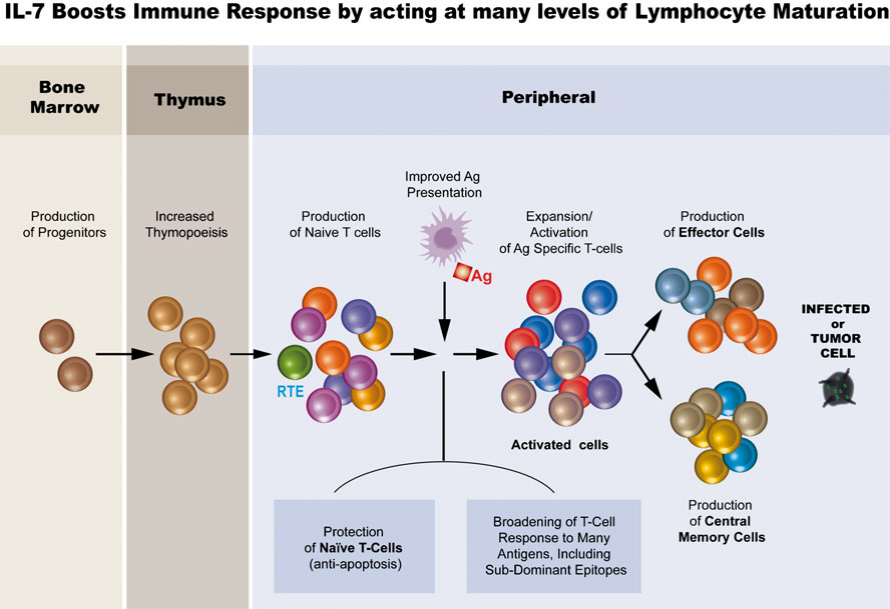
IL-7 a key factor in thymopoiesis and T cell periperal homeostasis. IL-7 receptor is expressed on common lymphoid progenitors (CLP) cells in the bone marrow and is essential for maintenance or progenitor pool for both B and T cells. During T cell development in the thymus IL-7 is involved at different stages of T cell proliferation and positive/negative selection. Following thymic export, recent emigrant T cells (RTE) are incorporated to the periphery. In the periphery, IL-7 is a major anti-apoptotic/survival factor of T cells through elevation of Bcl-2 expression. It also controls homeostatic and antigen-driven expansion of both CD4 and CD8 T cells. Its capacity to augment the immune response to weak or low affinity antigens will lead to the recognition of tumoral antigens. The Th1 response directly target tumoral cells. Following antigen driven expansion of activated T cells, a small population of effector cells become long-lived memory T cells expressing high levels of IL-7 receptor, the long-lasting anti-tumoral response. IL-7 by controlling thymopoiesis and peripheral homeostasis as well as antigen response is a critical factor for the immune system

Thus, IL-7 is seen as a critical factor for T cell production, maturation and expansion and therefore an ideal candidate for immune reconstitution. This non-redundant function of IL-7 for T cell production and maintenance is very similar to erythropoietin for red blood cells, PDGF for platelets and G-CSF for neutrophils, and provides a solid basis for the development of a therapeutic product (Fig. 1).

## IL-7 key preclinical results support its therapeutic use

### IL-7 efficiently triggers immune reconstitution in various models of lymphopenia

Very early in its development, IL-7 was successfully tested in various models of immune reconstitution. In mice, using a single injection of cyclophosphamide, Morisey et al. induced lymphopenia and showed that IL-7 administration resulted in an accelerated recovery of peripheral CD4+ and CD8+ T cell numbers in the spleen and lymph nodes [55]. Alpdogan et al. [56] demonstrated the immune reconstitution effects of IL-7 administration in a mouse model of allogeneic HSCT and Storek et al. did the same in a baboon model of autologous transplant [57]. This impressive immune reconstitution potential is also well documented in three reports on SIV-infected monkeys [58–60] and has more recently been shown to counteract interferon-induced lymphopenia [61]. In all these models, reconstitution involves both CD4 and CD8 lymphocytes as well as naïve and memory T cells.

### IL-7 does not induce an inflammatory cytokine storm syndrome

In all in vivo preclinical models tested, IL-7 administration did not produce the acute inflammatory cytokine release observed with various other cytokines. During the preclinical toxicology studies conducted in macaques at very high doses (up to 1 mg/kg twice a week), IL-7 administration produced a massive expansion of T cells and accompanying infiltration of all lymphoid tissues accomplished as a cold infiltration without signs of inflammation or manifestations of acute cytokine storm. If IL-7 induces a massive expansion in most T cell populations this proliferation appears unlikely to be accompanied by T cell activation. This very probably reflects the quick internalization of the IL-7 receptor after IL-7 interaction leading to a transient refractoriness of the T cell to further IL-7 stimulation [62]. As demonstrated by Henriques et al., this is a feature of IL-7 that is very consistent with the disappearance of CD127+ cells and their reappearance after 5 to 7 days as observed in early IL-7 pharmacodynamic and pharmacokinetic studies conducted in monkeys [62]. This lack of T cell over-activation also accounts for the possibility of producing viable models of transgenic mice that over express IL-7, something which is impossible with most activating cytokines such as IL-2, IL-12, and IL-15.

### IL-7 stimulates T cell homing to tissues

**Fig. 2:**
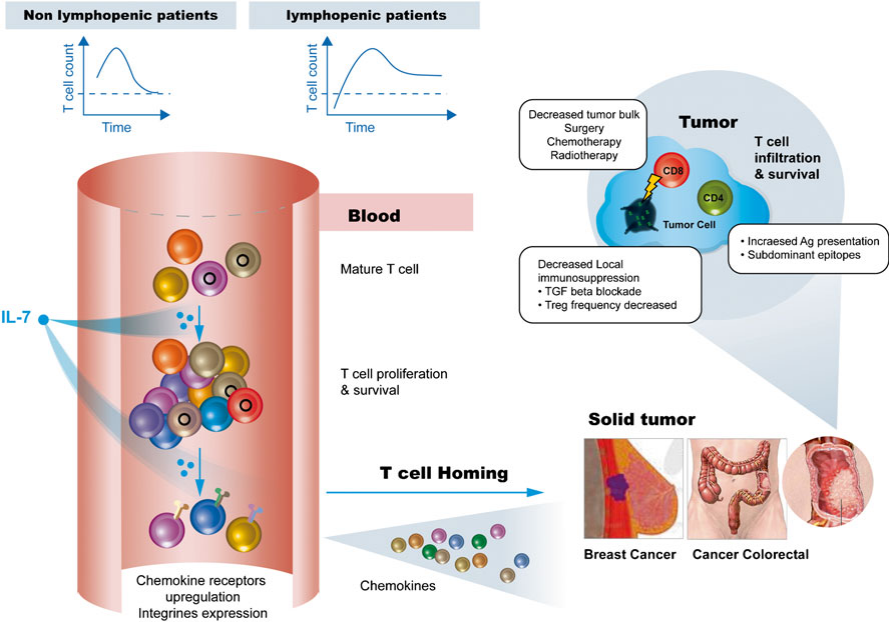
Possible mechanisms of action of IL-7 immunotherapy in oncology. IL-7 immunotherapy increases both CD4 and CD8 T cell subsets (Levy et al.). In non-lymphopenic recipients, this increase is transient and return to baseline (unpublished data CONVERT study); while in lymphopenic patients, a progressive restoration was observable with a sustained higher T cell count at 1 year post-treatment [74]. T-lymphocyte infiltration was demonstrated in lymph nodes, gut, and skin lymphoid tissue [60] in primates following IL-7 treatment. The cytokine lead to an upregulation of homing chemokine receptors on T cells including CXCR4, CCR6, and CCR9 coupled with increased chemokine levels in tissues (CCL19, CCL20, CCL21, and CCL25) and plasma (CCL3, CCL4, and CXCL12). The teams of Li et al. and Pellegrini et al., elegantly demonstrated that IL-7 treatment of mice induces a massive T cell infiltration in murine tumor leading to an enhanced anti-tumor protection correlated with an increased number of activated dendritic cells [61, 62]. Moreover, IL-7 will decrease the immunosuppressive environment in the tumor by decreasing TGFbeta secretion a key cytokine for immunosuppressive Treg. By increasing the number of tumor reactive T including cells with a memory phenotype cells and decreasing immunosuppression will result in a persistent and long-lasting anti-tumor T cell response

Using the rhesus macaque primate model to investigate the mechanisms involved in the short-lived, initial peripheral T cell depletion observed after IL-7 administration, Beq et al. [63] found that IL-7 administration induces a massive and rapid T cell migration from the blood into various organs, including the lymph nodes, parts of the intestine and the skin. This homing process is initiated after the induction of chemokine receptor expression by circulating T cells. In fact, all monkeys treated with IL-7 showed an increased expression of CD62L (L-selectin) and, when assessed, also showed an increase in α4β7 (MadCAM-1 integrin). Interestingly, in both anti-tumor responses discussed below, a massive tumor T cell infiltration was documented (Fig. 2).

### The simultaneous production of CD4 and CD8 T cells contributes to restoring or expanding efficient immune responses

Observations of this set of immunological effects lead to the further testing of IL-7 in various preclinical tumor models. In these studies, it rapidly became apparent that IL-7 alone does not seem to trigger anti-tumoral immune responses. Instead, any such response triggered by a vaccine, an adoptive T cell therapy, or any therapy able to stimulate antigen-presenting cells (APC) is amplified and prolonged by IL-7 treatment, leading to an improved outcome in the animal model. Two of these vaccine models seem particularly suited for further investigations in the clinical setting.

In combining IL-7 administration with a GM-CSF-secreting tumor cell immunotherapy directed against colon carcinoma or malignant melanoma, Li et al. observed an increased number of activated dendritic cells, T cells in lymphoid tissues, and an increase of activated effector T cells in the tumor microenvironment. Importantly, this observation correlates with a more potent systemic tumor-specific T cell response leading to prolonged survival of the tumor-bearing mice [64].

In a complex double-transgenic mouse model using LCMV infection as a vaccine against a spontaneous SV40-driven tumor mouse model, Pellegrini et al. observed that short term IL-7 therapy potently enhanced vaccine-mediated immunity, though it was inefficient in promoting antitumor immune responses in the absence of vaccination. To promote the anti-tumor response, IL-7 treatment was shown to antagonize various inhibitory networks, all of which are well documented at both the cellular and molecular level [65].

## IL-7 for immune reconstitution: clinical data

### The clinical development of recombinant human IL-7 follows two main pathways

In the setting of immunosuppression, usually associated with low to very low T cell counts, IL-7 is used for immune reconstitution. This setting includes HIV-infected patients who have not recovered adequate CD4 T cell counts after 1 or 2 years of HAART (the so-called “immune non-responders”), post-HSCT patients who have received a cord blood transplant or grafted with CD34+ cells (both very poor in lymphocytes progenitors), patients with rare cases of idiopathic lymphopenia, and approximately 25% of post-chemotherapy cancer patients. Soon, frequent cases of sepsis, where lymphopenia appears to be the main cause of the pathologic events leading to patient death, may also be classified in this group, along with patients exhibiting severe lymphopenia from any cause found in association with massive viral infections caused by pathogens usually controlled by a healthy immune system, including the EBV, CMV, HHV-6, and JC viruses.

Together, non-lymphopenic patients with chronic viral or bacterial infections or with cancer can benefit from IL-7 treatment if it is combined with an additional therapy utilized to create conditions favorable to the emergence of an immune response. This is exactly the case in chronic viral infections when a direct acting antiviral is used to decrease massive antigen load and thereby rescue some of the exhausted T cells expressing the PD1^+^ marker. This is also the case when another immune intervention such as a therapeutic vaccination is used to trigger the initiation of an immune response. Clinical studies in chronic HCV and HBV infections are currently ongoing to test this promising immune enhancement approach to IL-7 therapeutic use.

Rather than reviewing the IL-7 clinical effects by individual disease conditions, we will summarize below the main effects consistently observed in the clinical setting, illustrating the quality and the multiple dimensions of IL-7-induced immune reconstitution. This better supports and justifies the current therapeutic approach utilized in treating cancer patients with IL-7.

### Distinct differences in IL-2 and IL-7 effects on T cells explain the failure of IL-2 in immune reconstitution

In advocating the development of IL-7 for immune reconstitution, the failure of IL-2 therapy in two major studies focused on the immune reconstitution of HIV patients represents an instructive opportunity for review of the main differences between these two cytokines. Like IL-7, interleukin-2 is a key cytokine component of the immune system responsible for regulating tolerance and immunity [66]. In designing HIV studies for IL-2, its known capacity for inducing T cell proliferation, and specifically CD4 T cell expansion, made it a logical drug candidate for HIV patients who suffered from the loss of peripheral and mucosal CD4 T cells. Yet, in attempting to restore CD4 T cell populations through IL-2 administration, results from two pivotal clinical studies, SILCAAT for extremely lymphopenic HIV patients and ESPRIT for the less lymphopenic HIV patients, failed to demonstrate any clinical benefit from this therapy, although patients did exhibit increased CD4 T cell counts [67].

A detailed phenotypic analysis [68] of the CD4 produced by IL-2 treatment explains the failure of these two studies. IL-2 delivers essential signals for thymic development of regulatory T (Treg) cells and later in the periphery promotes their homeostasis and function [69]. Treg cells are increased in most human solid tumors [70]. In murine models, selective elimination of Treg cells (i.e., CD4 + CD25 + FOXP3+) restores an effective anti-tumor immune response leading to tumor regression. Treg cells are now increasingly being studied in the oncology field due to their apparent ability to limit the potency of the T cell response directed against tumors, either physiologically or following immunotherapy. Recent work has demonstrated that Treg cells selectively activated in the tumor contribute to tumor progression [71]. Thus, IL-2 expansion of CD4+ Treg cells in HIV patients could not be expected to cure the deficit in conventional T cells seen in this disease nor contribute to decreasing the incidence of opportunistic infections and AIDS or non-AIDS-related malignancies.

In fact, early transgenic models of IL-2 gene KO mice have shown, contrary to what is seen in the case of the IL-7 gene KO, that this does not lead to SCID syndrome but rather to the development of a fatal immunopathology characterized by lymphoadenopathy, splenomegaly, T cell infiltration of the bone marrow, loss of B cells, anemia, and inflammation of the gut [72]. Furthermore, studies of IL-2R beta gene KO mice demonstrate that IL-2R beta is required to keep the activation programs of T cells under control, to maintain homeostasis, and to prevent autoimmunity [73]. Table 1 summarizes the various features of IL-2 and IL-7 immune effects.

**Table 1:** Comparison of immune effects of IL-7 and IL-2

	IL-7	IL-2
Gene KO	Deletion of most T cells [43]	Hyper-proliferation of T cells, autoimmune syndrome [73]
Receptor gene KO confirmation	SCID syndrome in babies with mutation of CD127/IL-7R [44]	
Production of CD8 T cells	+++ Long lasting [80]	NO [68]
Production of suppressor Treg	Very low, decrease in Treg frequency [81]	High, increase in Treg frequency [68]
THYMUS and repertoire	Support of thymopoietic activity broadening of TCR repertoire [80, 82, 83]	NO thymic support [67]
Interaction with immunosuppressive TGFβ	Antagonism of TGFβ [77]	Synergy with TGFβ for Treg production [84]
Life of produced T cells	Long lasting through anti-apoptotic support [80, 82, 83]	Short
Clinical tolerance	Good: loss of IL-7R on activated T cells protects from over-activation [80, 82, 83]	Poor: risk of over-activation plus NK stimulation [67]

### Intensity: IL-7 therapy systematically and durably increases both CD4 and CD8 T cell counts

On the basis of data obtained from more than 200 patients treated with IL-7 in the clinical trials conducted by Cytheris, accumulated evidence indicates the potent lymphopoietic effect of this cytokine. The lymphopoietic response and peripheral expansion detected in all treated patients as a consequence of IL-7 therapy involves both CD4 and CD8 T cells. The CD4 T cells produced are conventional T cells with detectable helper function. Although IL-7 treatment induces a moderate increase in Treg, in relation to the increase of all other conventional T cells this increase actually translates into a decreased frequency of Treg. Interestingly, in HIV immune non-responders who have undergone several years of HAART therapy but whose CD4 counts remain below 250/mm^3^, a short 3-week cycle of IL-7 is sufficient to bring the CD4 counts of these patients above the threshold level of 500/mm^3^ and, furthermore, to maintain these CD4 counts above baseline levels for at least 1 year after this first cycle of IL-7 treatment. This long-lasting effect was also observed in lymphopenic patients, where the curve of T cell counts was observed to peak a few weeks after the IL-7 cycle, then drop to a plateau where it stabilized. The shape of this curve was different in non-lymphopenic patients, where the same treatment produced a temporary increase characterized by a dome shaped curve followed by a return to baseline after a few weeks.

Throughout the IL-7 dose escalation studies explored in various patient subpopulations, a dose-related effect from 3 to 10, 20, and 30 μg/kg was consistently observed. Considering the long-term maintenance of these effects, it appears that 10 and 20 μg/kg/week provide the best results together with an optimal clinical tolerance. This is consistent with the pharmacokinetic of the product which appears to be linear up to 20 μg/kg but becomes non-linear beyond this point, probably reflecting a saturation of the high-affinity target-mediated clearance at 30 μg/kg/week.

From the data accumulated in immune-suppressed patients it is apparent that the most lymphopenic benefit is obtained from the 20 μg/kg dose level, at least as a first induction cycle. More clinical results will be needed to determine the benefit of the 10 μg/kg/week dose for which only preliminary results are available from the less immunosuppressed patients. This dose will be explored to determine its utility in the maintenance cycle of patients who have already received a first IL-7 cycle and have recovered a CD4 count that is above 400/mm^3^ (Levy, Y ICAAC 2009 abstract 3585; Perales, M-A ASH 2011 abstract 674).

This stable and combined increase of both CD4 and CD8 T cells is promising because CD8 T cells are known to be the best effectors of immune response. Thus, for these cells to be fully functional and migrate to the tumor site where they can then express their cytolytic activity they require the efficient CD4 support in which IL-2 and IL-21 production appears essential.

### Quality and variety: IL-7 therapy impacts all T cells subtypes

**Fig. 3:**
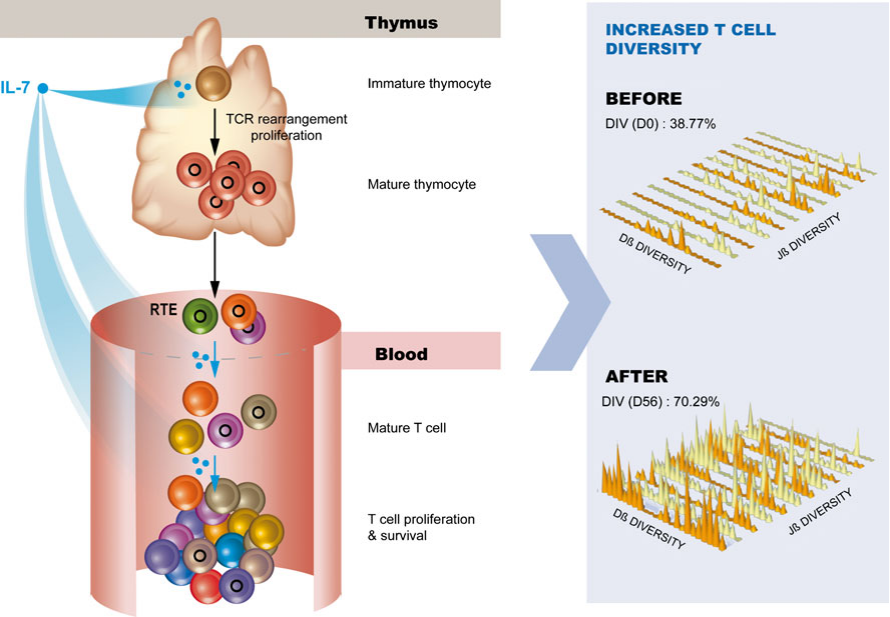
Impact of IL-7 on T cell repertoire. IL-7 has a major role in preserving the naïve T cell repertoire by increasing thymopoiesis and cell viability in absence of antigen stimulation. Thymopoiesis increase was demonstrated by the increase of TRECs following IL-7 immunotherapy in HIV patients. By inducing a higher rate of thymocyte proliferation, the cytokine generates more diverse TCR rearrangement contributing to an increase of RTE and naive T cell subsets in the periphery. An increase of the T cell pool diversity was demonstrated by TCR beta combinational diversity in HIV-infected patients treated with IL-7(manuscript in preparation INSPIRE 1 study). Peripheral expansion of pre-existing T cells probably contributes to the TCR repertoire diversification observation. By increasing thymus activity, output, and peripheral expansion of produced cells IL-7 potentially increase T cell repertoire diversity

IL-7 therapy increases both naive and memory T cells. The very significant increase of naïve T cells also involves recent thymic emigrants (CD31+ bright). This increase in thymic output was also observed by measuring the production of T cell receptor excision circles (TRECs; small DNA excision circles produced during the recombination of the TCR and reflecting the thymopoietic activity) and later through the measure of sj/βTRECs ratios, a marker reflecting the same effect but neutralizing the dilution due to the massive IL-7 T cell production (Fig. 3).

### IL-7 therapy also produced memory T cells, effectors, and importantly for long-term protection central memory T cells

All these effects were consistent with the known presence of the IL-7 receptor on T cells, representing the sub population with the most receptors, naïve, and central memory, being preferentially expanded.

The proliferative effect measured by Ki67 was obvious in the early days of treatment and the anti-apoptotic effect of IL-7 guaranteed the stability of these effects. The distribution of these various subpopulations did not differ significantly when measured just after the treatment cycle or a few months after.

### IL-7 therapy can increase the diversity of the T cell receptor repertoire: “the breadth dimension”

The observation of a thymopoietic effect confirmed earlier data sets produced in IL-7 treated monkeys as well as early thymic assessments performed on cancer patients. In addition to its lymphopoietic effect, IL-7 therapy can also raise clonal diversity by increasing the sensitivity of the TCR, not only to dominant epitopes, but also to sub-dominant epitopes as well [74–76]. The combination of these two effects results in an increased diversity of the TCR repertoire in treated patients. This was again assessed by utilizing the double PCR technology of ImmunID Technologies. The increase in TCR diversity has already been confirmed in HIV- and HCV-infected patients and after HSCT. These results also confirm previous data obtained by the immunoscope technology in cancer patients (Fig. 3). These increases in the diversity of the TCR repertoire are expected to increase the breadth of response against pathogens as well as against the escape of viruses by mutation and of malignant cells by immune editing.

### IL-7 therapy can increase the homing of T cells to lymphoid tissues and target organs

Supporting previous data from experimental monkey studies, the measure of CD62L and α4β7 on T cells after IL-7 treatment confirmed their potential for migration to tissues. In fact, due to the massive T cell depletion observed in HIV-infected patients and the key pathogenic role of this depletion in this disease and in the residual disease observed in immune non-responders to HAART, we were encouraged to measure the ability of IL-7 therapy to repopulate the gut with T cells. This was confirmed by gut biopsies performed on these patients, showing a significant repopulation in most patients after just one 3-week cycle of treatment. These investigations are still ongoing and are being extended to patients receiving more than one IL-7 cycle in order to explore the stability of this T cell repopulation.

## Some interesting facets of IL-7 activity remain to be confirmed and detailed at the clinical level

Though the expression of α4β7 allowing the extravasation of lymphocytes to the peripheral tissues certainly facilitates access to the tumor, fibrosis nevertheless remains a potential barrier, blocking access of T cells to their malignant targets. In his view, the IL-7 antagonism of TGF-β and the resulting block of fibrosis observed in an experimental model (bleomycin-induced lung fibrosis) could represent an interesting aspect of IL-7 activity which remains to be demonstrated in the clinic [77]. Antagonizing the immune-suppressive of TGF-β would also represent a positive trait of IL-7 anti-tumoral activity [78, 79].

Finally, the proliferative effects of IL-7 on the lymphocyte population are not limited to the αβT cells, as IL-7 also triggers the expansion of γδ T cells and invariant NKT. At the hinge between adaptive and innate immunity these cell populations certainly must participate in cancer immune surveillance, a feature that will be investigated in future clinical studies.

## Conclusions

While anemia and platelet loss are quickly apparent to the clinician and call for immediate therapeutic correction, the case of lymphopenia is more surreptitious. Behind an apparent normal standard hematology cell count, a critical deficit can be hidden involving a global deficit of T cells (CD3), helper cells (CD4), or an oligoclonality, also known as “divpenia” for the loss of diversity. Together, these conditions open gaps in the immune repertoire or result in a deficit of migration which leaves substantial room for severe local lymphopenia in the vicinity of the tumor.

**Fig. 4:**
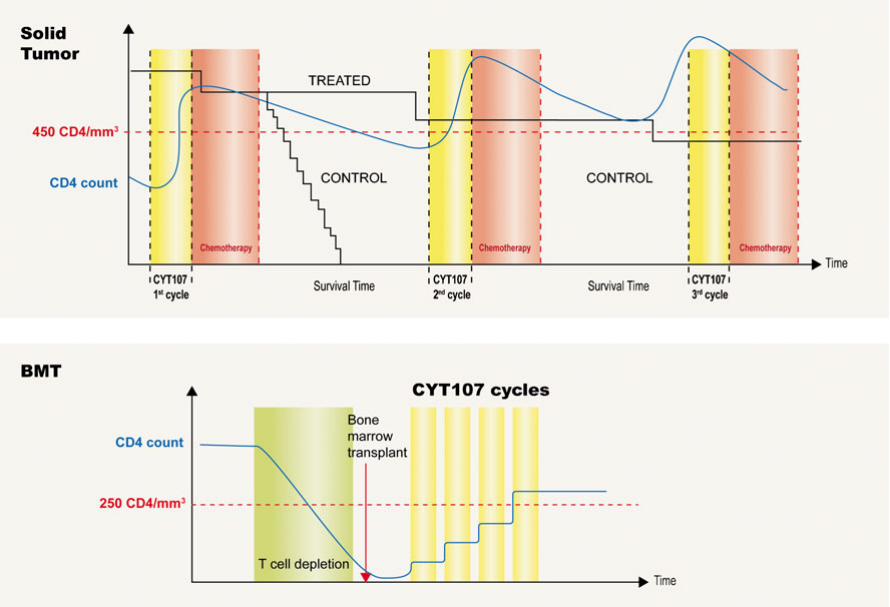
Clinical trial design involving IL-7 immunotherapy. As lymphopenia was identified as a prognosis factor for overall survival and tumor progression in patients with advanced cancer, cycles of IL-7 could be used before chemotherapy intervention to restore global immunity. Two different types of cancer are envisaged here: solid tumor and bone marrow transplantation. In metastatic patients presenting a chemotherapy-induced lymphopenia, cycle of IL-7 before or during chemotherapy will counteract the lymphopenia effect and preserve T cell survival. Following T cell depletion in bone marrow transplanted patients, multiple cycles of IL-7 will be necessary to recover a competent immune system and reach a less life-threatening window

All along the dimensions of lymphopenia, preclinical and clinical data have accumulated to demonstrate the ability of exogeneous IL-7 therapy to repair these multiple deficits. With the availability of the first clinical results, there is now a high probability for demonstrating a true clinical benefit of IL-7 treatment in the most lymphopenic patients. The development of IL-7 therapy for the prevention of opportunistic infections and relapses or incidence of new malignancies is ongoing in the setting of post-HSCT and HIV infection. With the production of new data indicating short term post-chemotherapy survival of cancer patients with lymphopenia characterized by a loss of CD4 T cells or repertoire diversity (divpenia), a new therapeutic paradigm is beginning to open based on the role of the immune system in treating these cancer patients (Fig. 4). Though it is now obvious that a thrombocytopenia can lead to death by fatal hemorrhage, until recently it was much less obvious that loss of lymphocytes could also lead to death by a breakdown in the immune barrier to infectious agents or malignant cells, including metastatic cells.

Finally, in the current acceleration in the development of anticancer immunotherapies, most efforts have been deployed to trigger anti-malignant cell responses, a difficult task against cells made invisible to the immune system. The concept of immunoediting has been instrumental in advancing our understanding of how an efficient immune response can be insufficient and unstable. As a counter balance, then, it has now become clear that the addition of IL-7 support could bring to these developing immunotherapeutic approaches, the intensity, breadth, and stability needed to eliminate the malignant process before the advent of an efficient escape facilitated by immunoediting.
